# Neuropsychological Functioning in Users of Serotonergic Psychedelics – A Systematic Review and Meta-Analysis

**DOI:** 10.3389/fphar.2021.739966

**Published:** 2021-09-16

**Authors:** Lukas A. Basedow, Thomas G. Riemer, Simon Reiche, Reinhold Kreutz, Tomislav Majić

**Affiliations:** ^1^ Department of Child and Adolescent Psychiatry, Faculty of Medicine, Technische Universität Dresden, Dresden, Germany; ^2^ Institute of Clinical Pharmacology and Toxicology, Corporate Member of Freie Universität Berlin, Humboldt-Universität zu Berlin, and Berlin Institute of Health, Charité–Universitätsmedizin Berlin, Berlin, Germany; ^3^ Department of Psychiatry and Psychotherapy, Corporate Member of Freie Universität Berlin, Humboldt-Universität zu Berlin, and Berlin Institute of Health, Charité–Universitätsmedizin Berlin, Berlin, Germany

**Keywords:** psychedelic, LSD, ayahuasca, peyote, attention, neuropsychology, inhibition, memory

## Abstract

**Background:** Serotonergic psychedelics (SPs) like LSD, psilocybin, DMT, and mescaline are a heterogeneous group of substances that share agonism at 5-HT_2a_ receptors. Besides the ability of these substances to facilitate profoundly altered states of consciousness, persisting psychological effects have been reported after single administrations, which outlast the acute psychedelic effects. In this review and meta-analysis, we investigated if repeated SP use associates with a characteristic neuropsychological profile indicating persisting effects on neuropsychological function.

**Methods:** We conducted a systematic review of studies investigating the neuropsychological performance in SP users, searching studies in Medline, Web of Science, embase, ClinicalTrials.gov, and EudraCT. Studies were included if they reported at least one neuropsychological measurement in users of SPs. Studies comparing SP users and non-users that reported mean scores and standard deviations were included in an exploratory meta-analysis.

**Results:** 13 studies (N = 539) published between 1969 and 2020 were included in this systematic review. Overall, we found that only three SPs were specifically investigated: ayahuasca (6 studies, *n* = 343), LSD (5 studies, *n* = 135), and peyote (1 study, *n* = 61). However, heterogeneity of the methodological quality was high across studies, with matching problems representing the most important limitation. Across all SPs, no uniform pattern of neuropsychological impairment was identified. Rather, the individual SPs seemed to be associated with distinct neuropsychological profiles. For instance, one study (*n* = 42) found LSD users to perform worse in trials A and B of the Trail-Making task, whereas meta-analytic assessment (5 studies, *n* = 352) of eleven individual neuropsychological measures indicated a better performance of ayahuasca users in the Stroop incongruent task (*p* = 0.03) and no differences in the others (all *p* > 0.05).

**Conclusion:** The majority of the included studies were not completely successful in controlling for confounders such as differences in non-psychedelic substance use between SP-users and non-users. Our analysis suggests that LSD, ayahuasca and peyote may have different neuropsychological consequences associated with their use. While LSD users showed reduced executive functioning and peyote users showed no differences across domains, there is some evidence that ayahuasca use is associated with increased executive functioning.

## Introduction

In the past 25 years, there has been a surge of new research focusing on the biological mechanisms of action of serotonergic psychedelics (SPs) like lysergic acid diethylamide (LSD), psilocybin, and N,N-dimethyltryptamine (DMT) (Kyzar et al., 2017) and their therapeutic potential in different psychiatric indications (Mertens and Preller, 2021). SPs are a heterogeneous group of substances that share certain characteristics, which can be characterized structurally, pharmacodynamically, and with regard to the phenomenology of the altered states of consciousness (ASC) that they facilitate.

Regarding their chemical structure, SPs can be divided into three subgroups (Nichols, 2018): *Tryptamines*, such as psilocybin, 5-methoxy-dimethyltryptamine (5-MeO-DMT) or DMT which is the main psychedelic ingredient of ayahuasca, an Amazonian concoction containing different plants; *ergoline derivatives*, which are more complex molecules on the basis of a tryptamine structure, such as LSD; and *phenethylamines*, such as mescaline, the main psychoactive component of different cacti like peyote and San Pedro. In addition to the examples given above there is a variety of novel synthetic SPs belonging to each group (Liechti, 2015), with 2,5-dimethoxy-4-bromophenethylamine (2C-B) currently being the most popular (Winstock et al., 2021).

Furthermore, all SPs share agonistic activity at the 5-HT_2a_ receptor (5-HT_2a_R), which appears to be critical for their psychoactive effects (Vollenweider et al., 1998), even though different SPs exhibit different binding affinities to 5-HT_2_aR. For instance, LSD and psilocin (the active metabolite of psilocybin) show a high affinity for 5-HT_2a_R (K_i_ = 2–4 nM and K_i_ = 15–25 nM respectively), DMT has a lower affinity (K_i_ = 127 nM), while mescaline has a comparatively low affinity (K_i_ = 550 nM) (Nichols, 2004; Keiser et al., 2009). On the other hand, it was shown that LSD and DMT were comparatively less selective for 5-HT_2a_R binding than psilocin (Ray, 2010), which in turn was less selective than mescaline or 2,5-Dimethoxy-4-methylamphetamine (DOM). In addition to differences in binding affinity, SPs also differ in selectivity and ligand efficacy. For instance, phenethylamines serve primarily as agonists at 5-HT_2a_R, whereas tryptamines and ergoline derivatives also show significant agonist activity at the 5-HT_1a_ receptor (5-HT_1a_R) (Halberstadt and Geyer, 2011). Data on ligand efficacy is relatively sparse, but most SPs are generally considered to be partial agonists rather than full agonists at the 5-HT2aR (Nichols, 2016), with only few synthetic, substituted tryptamines reaching full agonist status (Nichols, 2012). Finally, the intracellular pathways activated by 5-HT_2a_R agonism seem to be critically involved in the typical subjective effects of SPs (Vollenweider and Preller, 2020). This is evidenced by the existence of non-psychedelic 5-HT_2a_R agonists like lisuride, which differ from SPs in which intracellular pathways they activate (González-Maeso et al., 2007). Furthermore, individual SPs seem to activate unique transcription processes, differentiating SPs from one another (González-Maeso et al., 2003, 2007; Kurrasch-Orbaugh et al., 2003).

Despite some pharmacological differences, all SPs apparently show the ability to facilitate similar ASCs marked by striking changes in perception (e.g. pseudo-hallucinations, synesthesia), cognition, mood, and sense of self (Preller and Vollenweider, 2018; Swanson, 2018). In fact, two older studies indicate that the effects of psilocybin, LSD, and mescaline are not distinguishable in blinded laboratory conditions (Hollister and Hartman, 1962; Wolbach et al., 1962), and reports of SP-induced ASCs strongly overlap across substances (Zamberlan et al., 2018).

SPs are also unique with regard to the temporal dynamics of their effects, where acute (psychedelic states), subacute (“afterglow” phenomena), and long-term effects can be distinguished (Majić et al., 2015). Additionally, SPs have been associated with persisting changes in traits such as openness to experience, neuroticism, mindfulness, and optimism (Carhart-Harris et al., 2016; Erritzoe et al., 2018; Griffiths et al., 2018; Polito and Stevenson, 2019; Madsen et al., 2020). However, SP use has also been reported to exhibit prolonged negative consequences. Most prominent is an enduring psychotic reaction to SP use, which is probably rare but can occur even after a single administration in psychosis-prone individuals (Strassman, 1984). Another adverse reaction, which has been recognized early on in the field of psychedelic research, is the occurrence of short transient flashbacks or chronic and invasive perceptual distortions, known as hallucinogen persisting perception disorder (HPPD) (Halpern et al., 2016). Reports of HPPD are rare and most commonly related to the use of LSD, with only one case so far involving psilocybin and no reported cases involving ayahuasca or mescaline (Martinotti et al., 2018). Another group of persisting complications which might have been underestimated so far are symptoms from the dissociative spectrum, such as the depersonalization and derealization syndrome (Simeon et al., 2009), which may sometimes overlap with HPPD.

While many persisting psychological effects of SP use have been investigated (Aday et al., 2020), neuropsychological consequences remain underexplored. Even though acute effects of SPs include impairment of neuropsychological performance (Gouzoulis-Mayfrank et al., 2002, 2006; Bouso et al., 2013; Barrett et al., 2018; Pokorny et al., 2019; Healy, 2021), so far only one systematic review has investigated persisting effects of SP use on neuropsychological functioning (Halpern and Pope, 1999). Although no residual neuropsychological consequences were identified, the authors point out that all of the studies included in their review exhibited methodological limitations, rendering their conclusions tentative.

Based on the evidence that SPs show the ability to facilitate different subacute and persisting psychological changes and given the renewed scientific and clinical interest in SPs, we aim to investigate if repeated SP use is associated with changes in neuropsychological performance. We explore this topic by conducting a systematic review of the literature and an exploratory meta-analysis of neuropsychological test outcomes.

## Methods

### Search Strategy

This review is reported according to the PRISMA statement (Moher et al., 2009). We performed electronic searches in Medline, Web of Science, and embase, from the respective database inception to November 18, 2020. Additionally, we searched the clinical trial registries ClinicalTrials.gov and EudraCT. The search was conducted using an algorithm connecting a selection of SPs and terms associated with neuropsychological testing or domains (shown in Supplemental Table S1) in an iterative manner. Given the broad variety of different available SPs, only those substances were included that exhibit a relevant degree of popularity and use prevalence in the population, such as the three most commonly used SPs, LSD, psilocybin and 2C-B (Evens et al., under review). Since we did not expect to find many relevant studies and because of the similarities in subjective experience and acute neuropsychological effects across substances discussed above, we decided to extend our search to all SPs across chemical sub-groups. References were retrieved through the electronic searches and by manual searches through the reference lists of review articles. All articles published in English, German, French, or Spanish were included. The PICOS (population, intervention, comparisons, outcomes, study type) selection criteria for our search are described in the supplementary methods section.

### Data Extraction

All search results were screened independently by two researchers (LAB, TGR), while a third (TM) provided input if it was not clear whether an article should be included or excluded. From the selected articles we recorded authors’ names, year of publication, duration and frequency of drug exposure, drug dosages, sample size, participant characteristics (number of female and male participants, mean age, age range), neuropsychological tests that were employed, and their results. In cases where data was missing for a study to be included in the meta-analysis, we contacted the authors. According to the content of the studies that were detected, the sample of studies was then divided into groups by substance, resulting in four groups: “LSD”, “Ayahuasca/DMT”, “Peyote/Mescaline”, and “Not specified”. Finally, the neuropsychological tests used in the studies were categorized into six domains: Memory, Executive Functioning, Attention, Visuospatial Abilities, Intelligence, and Other.

### Study Quality

We used the Newcastle-Ottowa-Scale (NOS) (Wells et al., 2000) to estimate study quality and the risk of bias. The scale assigns a score for three parameters: “selection”, “comparability”, “outcome”, with a maximum total score of 9. We considered a study to be of high quality if it fulfilled both comparability criteria and reached a total score of seven or higher. LAB and TGR rated study quality independently, and subsequently formed a consensus on the rating of each parameter. Discrepancies were solved with input from TM.

### Meta-Analysis

Studies that reported results as mean scores with standard deviations were eligible for meta-analysis. For each neuropsychological test and subtest, a test of overall effect across means was conducted if at least three studies were available reporting mean and standard deviation for that test. In cases where different studies reported different outcome measures for the same test (for example, number of errors vs reaction time), the overall effect for the standardized mean difference was calculated instead. All calculations were performed with Review Manager 5.3 (The Cochrane Collaboration, 2014). In line with previous recommendations, we did not adjust *p*-values for multiple comparisons, due to the exploratory nature of our study (Rothman, 1990). We intended to perform four a-priori planned sensitivity analyses: 1) restriction to the studies with a rating of seven or higher on the NOS, 2) selective inclusion of studies employing matched control groups, 3) restriction to those studies examining the same SP, and 4) exclusion of the study with the greatest weight. In addition, heterogeneity in effect sizes was assessed using the I^2^ statistic.

## Results

### Study Selection

**FIGURE 1:**
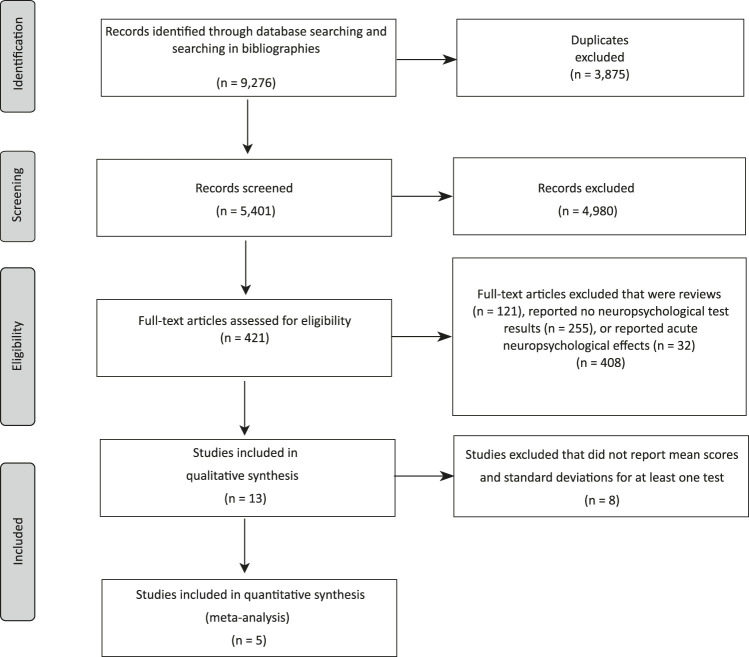
PRISMA Flow diagram.

Excluding duplicates, our search identified 5,401 articles. Of these, 4,980 were rejected after title and abstract screening for not dealing with the effects of SPs on neuropsychological functioning, leaving 421 articles for full-text screening. After subsequent full-text screening, a total of 13 studies were left for inclusion in the systematic review (Cohen and Edwards, 1969; McGlothlin et al., 1969; Wright and Hogan, 1972; Culver and King, 1974; Matefy et al., 1979; Vardy and Kay, 1983; Grob et al., 1996; Doering-Silveira et al., 2005b; Halpern et al., 2005; Bouso et al., 2012, 2015; Barbosa et al., 2016; Kaasik and Kreegipuu, 2020). Five of these articles met the criteria for inclusion in the meta-analysis (Grob et al., 1996; Doering-Silveira et al., 2005b; Halpern et al., 2005; Bouso et al., 2012; Barbosa et al., 2016). Details of the different phases of the search are shown in Figure 1.

All included studies were cross-sectional studies, comparing cohorts of users of SPs (*n* = 539) to various groups of non-users. Five of the selected studies investigated the effects of repeated LSD use (*n* = 101) (Cohen and Edwards, 1969; McGlothlin et al., 1969; Wright and Hogan, 1972; Culver and King, 1974; Vardy and Kay, 1983), six explored the effects of ayahuasca (*n* = 343) (Grob et al., 1996; Doering-Silveira et al., 2005b; Bouso et al., 2012, 2015; Barbosa et al., 2016; Kaasik and Kreegipuu, 2020), one dealt with peyote users (*n* = 61) (Halpern et al., 2005), and one study did not specify which SPs had been used by the participants (*n* = 34) (Matefy et al., 1979). The participant demographics, study characteristics, and neuropsychological tests results for all included studies are shown in Table 1.

**TABLE 1:** Overview of all included studies and comparisons.

Study	Serotonergic psychedelic		Controlled for other substance use	Sample characteristics	Assessed neuropsychological domains					
	Substance	# Of exposures		N (female), age	MEM	EXE	ATT	VSA	INT	OTH
Barbosa et al. (2016)	Ayahuasca	Median 150	No	Users: 30 (14), Median 42.5. Non-Users: 27 (13), Median 45	CVLT interference list**: users > non-users (*p* < 0.05), 14 other measures n.s	Stroop 4 measures n.s., TMT-B n.s	CPT 12 measures n.s., TMT-A n.s	ROCF 3 measures n.s	—	—
Bouso et al. (2012)–First assessment	Ayahuasca	Range 60–1,440	No	Users: 128 (68), Mean 36.7. Non-Users: 115 (60), Mean 35.9	—	Stroop word list**, color list**, interference effect**; WCST total, preservative errors**, non-preservative errors**, total errors: users**, 1 other measures, n.s.; LN-Sequencing**	—	—	—	—
Bouso et al. (2012)–Second assessment	Ayahuasca	Range 360–1,440	No	Users: 78 (38), Mean: 39. Non-Users: 68 (42), Mean: 37.7	—	Stroop word list**; 3 other measures, n.s.; WCST total**, non-preservative errors**; 3 other measures, n.s.; LN-Sequencing**	—	—-	—	—
Bouso et al. (2015)	Ayahuasca	Range 50–352. Mean 123	Yes	Users: 22 (16), Mean: 40.9. Non-Users: 22 (16), Mean: 41.5	Two-back task hits**, misses**, reaction time on hits**, a-prime**, d-prime**; 3 other measures, n.s	WCST 4 measures, n.s.; Task-switching % correct non-switch**, % error non-switch**; 4 other measures, n.s	—	—	—	—
Doering-Silveira et al. (2005b)	Ayahuasca	Range 24–open	No	Users: 40 (18), Mean: 16.5. Non-Users: 40 (18), Mean: 16.6	WAIS digit span 3 measures, n.s.; UCLA AVLT trial 2**, trial 4**, total 1–5**; 6 other measures, n.s	TMT-B, n.s.; WAIS digit symbol, n.s.; Stroop 3 measures, n.s	CPT 8 measures, n.s.; TMT-A, n.s	ROCF 2 measures, n.s	—	WAIS (object assembly + symbol search) 2 measures, n.s
Grob et al. (1996)	Ayahuasca	Range 240–open	No	Users: 15 (0), -Non-Users: 15 (0), -	UCLA AVLT trial 5**; 4 other measures, n.s	—	—	—	—	—
Kaasik & Kreegipuu (2020)	Ayahuasca	Range 1–250. Median 10	—	Users: 30 (15), Mean: 38.7	—	—	—	—	Raven matrices, n.s	—
Cohen & Edwards (1969)	LSD	Median70	No	Users: 30 (15), mean: 21.7 Non-users: 30 (15), mean: 21.8	—	TMT-B, n.s	TMT-A*	HR tactual performance, n.s.; spatial orientation*	Raven matrices, n.s	HR category, n.s.; speech perception, n.s.; finger tapping, n.s.; rhythm, n.s
Culver and King, (1974)	LSD or Mescaline	Range 12–58. Mean 22.8. Median 17	Yes	Users: 14 (-), -Non-Users: 14 (-)	WAIS digit span, n.s	WAIS block design, n.s.; WAIS picture arrangement, n.s.; TMT-B*	TMT-A*	WAIS picture completion, n.s.; HR tactual performance: 3 measures, n.s.; HR speech sounds perception, n.s.; HR tactual form board, n.s.; HR hidden patterns, n.s.; HR cube comparisons, n.s	WAIS (verbal + performance + full scale): 3 measures, n.s	WAIS: 6 remaining measures, n.s
Halpern et al. (2005)	Peyote	Range 150–500. Median 300	Yes	Users: 61 (46), Median: 31. Non-Users: 79 (65), Median: 29	WAIS digit span: 3 measures, n.s.; Wechsler memory scale: 2 measures, n.s	TMT-B: 2 measures, n.s.; Stroop: 6 measures, n.s.; WAIS block design, n.s.; WAIS digit symbol, n.s.; WCST: 6 measures, n.s	Auditory CPT: 3 measures, n.s.; TMT-A: 2 measures, n.s	ROCF: 3 measures, n.s	Raven matrices, n.s	—
Matefy et al., (1979)	Not specified	No information	No	Users: 34 (-), -Users with flashbacks: 29 (-), -Non-Users: 24 (-), -	—	—	Self-designed reaction time task: 5 measures**, 3 measures, n.s	—	—	-
McGlothlin et al, (1969)	LSD	Range 20–1,100. Median 75	No	Users: 16 (4), Mean: 40. Non-Users: 16 (4), Mean: 40	—	TMT-B, n.s	TMT-A, n.s	Spatial orientation, n.s.; MPDT, n.s.; Porteus Maze, n.s.; Embedded figures, n.s	Shipley-Hartford test 3 measures, n.s	Associational fluency, n.s.; HR finger tapping 2 measures, n.s.; HR rhythm, n.s.; HR category*
Vardy and Kay, (1983)	LSD	No information	No	Users: 21 (4), Mean: 19.8 Acute, non-using, schizophrenics: 21 (13), Mean: 20.7	—	—	—	Bender-Gestalt test, n.s., Benton test 2 measures, n.s	WAIS (verbal + performance + full scale): 3 measures, n.s	—
Wright and Hogan, (1972)	LSD	Range 5–100 Mean 29.3	No	Users: 20 (5), Mean: 20.2. Non-Users: 20 (5), Mean: 20.2	WAIS digit span, n.s	WAIS digit symbol, n.s.; WAIS block design, n.s.; WAIS picture arrangement, n.s.; TMT-B, n.s	TMT-A, n.s	WAIS picture completion, n.s. HR tactual performance 3 measures, n.s	WAIS (verbal + performance + full scale) 3 measures, n.s	WAIS information**; WAIS comprehension*; WAIS 4 remaining measures, n.s.; HR 6 measures, n.s

n.s., no significant differences between groups; * users < non-users; ** users > non-users; N*, sample size; *MEM*, memory; *EXE*, executive functions; *ATT*, attention; *VSA*, visuospatial abilities; *OTH*, other; *CVLT*, California Verbal Learning Test; *TMT*, Trail Making Test; *ROCF*, Rey-Osterrieth Complex Figure Test; *WCST*, Wisconsin Card Sorting Test; *LN*, letter-number; *WAIS*, Wechsler Adult Intelligence Scale; *UCLA AVLT*, University of California Los Angeles Auditory Verbal Learning Test; *CPT*, Continuous Performance Task*

### Study Quality

The median NOS score across all studies was 5, with one study receiving the lowest score of 3 (Kaasik and Kreegipuu, 2020) and one study receiving the highest score of 7 (McGlothlin et al., 1969) but not achieving a full comparability score, meaning no included study was rated as having high quality. The most common sources of bias were lack of an objective verification of SP exposure, with no studies providing this, and reduced comparability by not controlling for other substance use, with only three studies fulfilling this requirement (Culver and King, 1974; Halpern et al., 2005; Bouso et al., 2015). An overview of the complete ratings is given in Supplementary Table S2.

### Qualitative Analysis

#### Memory

Working memory was assessed using the digit span task of the Wechsler Adult Intelligence Scale (WAIS) in four studies (Wright and Hogan, 1972; Culver and King, 1974; Doering-Silveira et al., 2005b; Halpern et al., 2005) and the two-back task in one study (Bouso et al., 2015). Altogether, the samples included 157 users of SPs (34 LSD, 62 ayahuasca, 61 peyote) and 175 non-using controls. A significant difference was reported only in the two-back task (Bouso et al., 2015), with the ayahuasca users reaching a higher number of hits, a lower number of misses, and faster reaction times on hit trials than the non-using group. However, there was no difference between the groups in the rate of false alarms or the number of correct rejections.

Episodic memory was assessed with word list tasks (the UCLA Auditory Verbal Learning Test (UCLA AVLT) and the California Verbal Learning Task (CVLT) in three studies (Grob et al., 1996; Doering-Silveira et al., 2005b; Barbosa et al., 2016) and the immediate and delayed visual recall tasks from the WAIS in one study (Halpern et al., 2005). In total, 146 SP users were included (85 ayahuasca, 61 peyote) and compared to 161 non-users. One study (Grob et al., 1996) found that the ayahuasca users could recall more words on the fifth learning trial. However, there was no difference in total number of words recalled, in number of false positives, in words recalled after interference, or in words recalled after a delay. In another study (Doering-Silveira et al., 2005b), the ayahuasca-using group could recall more words on trials 2 and 4, and overall in the UCLA AVLT. On trials 1, 3, 5, 6, 7, 8, and 9, the groups showed no difference in performance. Finally, one study (Barbosa et al., 2016) reported that ayahuasca users performed better on the interference trial, but there was no significant difference in any other measure (performance on trials 1–5; sum score of trials 1–5; sum of intrusions over trials 1–5; short delay free recall; short delay cued recall; long delay free recall; long delay cued recall; total number of intrusions or recognitions; proactive inference score).

To date, no studies have assessed whether SP users differ from the non-using population in their long-term memory. Working memory performance as assessed by the two-back task and the WAIS digit span task does not appear to be related to regular use of SPs. Similarly, there are no consistent results pointing to a difference in performance on the verbal learning tasks. Generally, users of SPs performed better on some trials, but the trials in which performance differed were different in each study, indicating no clear pattern of results.

#### Executive Functions

Executive functions were assessed by seven different tasks (letter-number sequencing tasks, WAIS digit symbol, WAIS block design, WAIS picture arrangement, Stroop task, Wisconsin Card Sorting Task (WCST), and the set-shifting subtest of the Test of Attentional Performance (TAP)) in a total of nine studies (Cohen and Edwards, 1969; McGlothlin et al., 1969; Wright and Hogan, 1972; Culver and King, 1974; Doering-Silveira et al., 2005b; Halpern et al., 2005; Bouso et al., 2012, 2015; Barbosa et al., 2016). Overall, 339 SP users were included (198 ayahuasca, 14 LSD or mescaline, 61 peyote, 66 LSD) and compared to 355 non-users. Three of the studies (Culver and King, 1974; Bouso et al., 2012, 2015) reported significant differences in executive functioning, whereas no difference was reported in the other studies (Cohen and Edwards, 1969; McGlothlin et al., 1969; Wright and Hogan, 1972; Doering-Silveira et al., 2005b; Halpern et al., 2005; Barbosa et al., 2016).

Culver and King (1974) found that LSD users performed worse on a letter-number sequencing task (Trail Making Test B, TMT-B), while Bouso et al. (2012) found that their sample of ayahuasca users performed better than non-using controls in the same task. Additionally, Bouso et al. (2012) found that ayahuasca users performed better on the congruent word and color lists and the incongruent list in the Stroop task and made fewer errors overall and fewer non-perseverative errors on the WCST. No difference was observed in the number of perseverative errors or the number of achieved categories in the WCST. Bouso et al. (2015) found that the ayahuasca users made a higher number of correct non-switch decisions and a lower number of non-switch decision errors in the set-shifting task. Nevertheless, the groups did not differ in the number of correct switching decisions, in the number of switch errors, or their reaction times.

One study (Culver and King, 1974) reported lower performance in users of SPs on the TMT-B, while Bouso et al. (2012) observed the opposite pattern in their sample on a similar task. No other study that used this task detected any difference in performance between users and non-users. This holds similarly for the other tasks that assess executive functioning. Although Bouso et al. (2012) reported that ayahuasca users performed better on the WCST, this pattern was not observed by Bouso et al. (2015) or Halpern et al. (2005).

#### Attention

Attention was assessed via three different tasks in seven studies. All seven studies employed trial A of the Trail Making Test (TMT-A) (Cohen and Edwards, 1969; McGlothlin et al., 1969; Wright and Hogan, 1972; Culver and King, 1974; Doering-Silveira et al., 2005b; Halpern et al., 2005; Barbosa et al., 2016), three studies used the continuous performance task (Doering-Silveira et al., 2005b; Halpern et al., 2005; Barbosa et al., 2016), and one study used a self-designed simple reaction task (Matefy et al., 1979). Overall, 274 SP users were included (70 ayahuasca, 61 peyote, 80 LSD, 63 not specified) and compared to 250 non-users. Two of the studies (Cohen and Edwards, 1969; Culver and King, 1974) found that LSD users performed worse than non-using controls in TMT-A, and (Matefy et al., 1979) reported that the SP users did perform faster on the self-designed reaction time task than non-users. None of the other studies reported significant differences in an attention task (McGlothlin et al., 1969; Wright and Hogan, 1972; Doering-Silveira et al., 2005b; Halpern et al., 2005; Barbosa et al., 2016).

#### Visuospatial Abilities

Visuospatial and perceptual abilities were evaluated using eleven different tests (the Rey-Osterrieth Complex Figure task (ROCF), the Minnesota Percepto-Diagnostic Tests, the Porteus Maze, the embedded figures task, a map-reading task, the WAIS picture completion task, the Bender-Gestalt test, the Benton test, and the tactual performance, spatial orientation hidden pattern, and cube comparison subtests from the Halstead-Reitan battery (HR)) in eight studies (Cohen and Edwards, 1969; McGlothlin et al., 1969; Wright and Hogan, 1972; Culver and King, 1974; Vardy and Kay, 1983; Doering-Silveira et al., 2005b; Halpern et al., 2005; Barbosa et al., 2016). Overall, 232 SP users were included (70 ayahuasca, 61 peyote, 101 LSD) and compared to 246 non-users. Cohen and Edwards (1969) found reduced performance for LSD users in the HR spatial orientation task. No other studies detected differences in any of the tests.

#### Intelligence

Three measures of intelligence (Shipley-Hartford test, WAIS, Raven matrices) were used across six studies (Cohen and Edwards, 1969; McGlothlin et al., 1969; Wright and Hogan, 1972; Vardy and Kay, 1983; Halpern et al., 2005; Kaasik and Kreegipuu, 2020). Overall, 178 SP users were included (30 ayahuasca, 61 peyote, 87 LSD) and compared to 196 non-users. No significant differences were observed.

#### Other Measures

From the HR and WAIS test batteries eleven other subtests were included (HR: finger tapping, rhythm discrimination, category, speech perception; WAIS: information, comprehension, arithmetic, similarities, vocabulary, object assembly, and symbol search) in five studies (Cohen and Edwards, 1969; McGlothlin et al., 1969; Wright and Hogan, 1972; Culver and King, 1974; Doering-Silveira et al., 2005b). Overall, 120 SP users were included (40 ayahuasca, 80 LSD) and compared to 120 non-users. In three of the subtests, significant differences were found. McGlothlin et al. (1969) reported that LSD users performed worse in the category subtest, while Wright and Hogan (1972) observed that LSD users performed better on the information subtest and worse on the comprehension subtests than controls. McGlothlin et al. (1969) additionally administered an associational fluency task, while Culver and King (1974) included a laterality discrimination task, asked participants to fold paper in specific patterns, and recorded performance on a hand dynamometer. On none of these tests did they detect any difference.

### Quantitative Analysis

A complete overview of the measures on which a meta-analysis was performed, the number of included participants, the statistical method used, heterogeneity, and the effect estimates can be found in Tables 2–5. Forest plots for all performed analyses are shown in Supplementary Figures S1–4.

**TABLE 2:** Overview of means, mean differences and *p*-values on the Trail-Making Test.

Study	Serotonergic psychedelic		Control		Weight	Mean difference
	Mean (SD)	N	Mean (SD)	N		
**TMT-A (*p = 0.*98; I** ^**2**^ **= 9%)**						
Barbosa et al. (2016)*	26.0 (6.92)	30	28.07 (7.53)	27	25.3%	−2.07 [−5.84, 1.70]
Doering-Silveira et al. (2005a)	29.2 (8.86)	40	27.25 (8.26)	40	25.5%	1.95 [−1.80, 5.70]
Halpern et al. (2005)	23.9 (8.3)	61	23.9 (7.0)	78	49.3%	0.00 [−2.60, 2.60]
**TMT-B (*p = 0.*99; I** ^**2**^ **= 57%)**						
Barbosa et al. (2016)*	57.96 (14.03)	30	66.44 (21.63)	27	29.2%	−8.48 [−18.06, 1.10]
Doering-Silveira et al. (2005b)	61.38 (25.1)	40	56.0 (15.82)	40	30.4%	5.38 [−3.81, 14.57]
Halpern et al. (2005)	63.6 (20.6)	61	61.6 (16.9)	78	40.4%	2.00 [−4.39, 8.39]

* = Means and standard deviation courtesy of Barbosa et al.; *N*, sample size; *SD*, standard deviation; *TMT*, TrailMaking Test

**TABLE 3:** Overview of means, mean differences and *p*-values on the Rey-Osterrieth Complex Figure Task.

Study	Serotonergic psychedelic		Control		Weight	Mean difference
	Mean (SD)	N	Mean (SD)	N		
**ROCF–Copy (*p = 0.*26, I** ^**2**^ **= 57%)**						
Barbosa et al. (2016)*	33.05 (3.07)	30	33.444 (2.77)	27	11.9%	−0.39 [−1.91, 1.12]
Doering-Silveira et al. (2005a)	34.64 (1.46)	40	34.08 (2.58)	40	32.6%	0.56 [−0.36, 1.48]
Halpern et al. (2005)	33.9 (2.1)	61	33.6 (2.1)	78	55.5%	0.30 [−0.40, 1.00]
**ROCF–Delayed recall (*p* = 0.14; I** ^**2**^ **= 0%)**						
Barbosa et al. (2016)*	22.88 (7.51)	30	21.648 (6.03)	27	15.6%	1.23 [−2.29, 4.75]
Doering-Silveira et al. (2005b)	21.89 (5.0)	40	21.69 (6.79)	40	28.2%	0.20 [−2.41, 2.81]
Halpern et al. (2005)	22.9 (5.7)	61	21.5 (5.3)	78	56.2%	1.40 [−0.45, 3.25]

* = Means and standard deviation courtesy of Barbosa et al.; *N*, sample size; *SD*, standard deviation; *ROCF*, Rey-Osterrieth Complex Figure Test

**TABLE 4:** Overview of means, mean differences and *p*-values across Verbal Learning Tasks.

Study	Serotonergic psychedelic		Control		Weight	Mean difference
	Mean (SD)	N	Mean (SD)	N		
**VLT**–**Long delayed recall (*p = 0.*94; I**^**2**^**= 43%)**						
Barbosa et al. (2016)*	12.58 (2.8)	29	13.0 (2.09)	27	16.5%	−0.42 [−1.71, 0.87]
Doering-Silveira et al. (2005a)	12.4 (1.93)	40	12.93 (1.55)	40	30.9%	−0.53 [−1.30, 0.24]
Grob et al. (1996)	9.53 (2.64)	15	8.41 (1.62)	15	12.2%	1.12 [−0.45, 2.69]
Halpern et al. (2005)	12.2 (1.4)	61	11.9 (1.9)	78	40.4%	0.30 [−0.25, 0.85]
**VLT**–**Trial 5 (*p = 0.*68; I**^**2**^**= 69%)**						
Barbosa et al. (2016)*	13.17 (2.37)	29	13.44 (1.93)	27	32.3%	−0.33 [−1.03, 0.37]
Doering-Silveira et al. (2005b)	12.9 (1.5)	40	13.23 (1.72)	40	40.8%	−0.27 [−1.40, 0.86]
Grob et al. (1996)	11.21 (1.93)	15	9.5 (2.07)	15	26.8%	1.71 [0.28, 3.14]
**VLT**–**Short recall after interference (*p = 0.*93; I**^**2**^**= 57%)**						
Barbosa et al. (2016)*	12.17 (2.76)	30	12.37 (2.20)	27	31.5%	−0.20 [-1.49, 1.09]
Doering-Silveira et al. (2005a)	12.15 (1.9)	40	12.83 (1.58)	40	45.0%	−0.68 [−1.45, 0.09]
Grob et al. (1996)	9.53 (2.72)	15	8.16 (1.99)	15	23.4%	1.37 [−0.34, 3.08]
**VLT–Recognition (*p = 0.*84; I** ^**2**^ **= 31%)**						
Barbosa et al. (2016)*	15.25 (1.03)	29	15.19 (0.96)	27	32.1%	0.06 [−0.46, 0.58]
Doering-Silveira et al. (2005b)	14.55 (0.82)	40	14.78 (0.62)	40	55.6%	−0.23 [−0.55, 0.09]
Grob et al. (1996)	14.3 (0.72)	15	13.75 (1.76)	15	12.3%	0.58 [−0.38, 1.54]

* = Means and standard deviation courtesy of Barbosa et al.; *N*, sample size; *SD*, standard deviation; *VLT*, Verbal Learning Task

**TABLE 5:** Overview of means, mean differences and *p*-values on the Stroop Task.

Study	Serotonergic psychedelic		Control		Weight	Mean difference
	Mean (SD)	N	Mean (SD)	N		
**Stroop - Word list (*p = 0.*85; I** ^**2**^ **= 86%)**						
Barbosa et al. (2016)*	94.25 (14.49)	29	99.41 (15.49)	27	18.2%	−0.34 [−0.87, 0.19]
Bouso et al. (2012)–Jungle sample	86.36 (17.95)	56	77.38 (19.49)	56	20.5%	0.48 [0.10, 0.85]
Bouso et al. (2012)–Urban sample	94.11 (16.78)	71	82.27 (15.62)	56	20.7%	0.72 [0.36, 1.08]
Doering-Silveira et al. (2005a)	−16.2 (3.89)	40	−14.75 (3.34)	40	19.5%	−0.40 [−0.84, 0.05]
Halpern et al. (2005)	(−51.1) 9.8	61	−48.4 (8.4)	78	21.0%	−0.30 [−0.63, 0.04]
**Stroop**–**Color list (*p = 0.*24; I**^**2**^**= 82%)**						
Barbosa et al. (2016)*	74.21 (9.13)	29	72.74 (12.50)	27	17.9%	0.13 [−0.39, 0.66]
Bouso et al. (2012)–Jungle sample	62.2 (12.08)	56	57.09 (12.58)	56	20.6%	0.41 [0.04, 0.79]
Bouso et al. (2012)–Urban sample	69.27 (15.25)	71	55.09 (14.11)	56	20.7%	0.95 [0.58, 1.33]
Doering-Silveira et al. (2005b)	−13.03 (2.27)	40	−12.6 (2.22)	40	19.5%	−0.19 [−0.63, 0.25]
Halpern et al. (2005)	−62.8 (10.4)	61	−62.0 (10.7)	78	21.3%	−0.08 [−0.41, 0.26]
**Stroop**–**Incongruent list (*p = 0.*03; I**^**2**^**= 73%)**						
Barbosa et al. (2016)*	48.13 (8.29)	29	44.52 (7.48)	27	16.8%	0.45 [−0.08, 0.98]
Bouso et al. (2012)–Jungle sample	44.36 (18.81)	56	34.25 (8.68)	56	20.7%	0.69 [0.30, 1.07]
Bouso et al. (2012)–Urban sample	45.87 (13.78)	71	36.02 (11.7)	56	21.3%	0.76 [0.40, 1.12]
Doering-Silveira et al. (2005a)	−24.95 (7.14)	40	−25.05 (8.0)	40	19.2%	0.01 [−0.43, 0.45]
Halpern et al. (2005)	−115.1 (22.6)	61	−114.7 (23.2)	78	22.0%	−0.02 [−0.35, 0.32]

* = Means and standard deviation courtesy of Barbosa et al.; *N*, sample size; *SD*, standard deviation

#### Memory

Verbal Learning task*.* In the different verbal learning tasks, four studies could be compared on delayed recall ability (Grob et al., 1996; Doering-Silveira et al., 2005b; Halpern et al., 2005; Barbosa et al., 2016). The four studies had 305 participants in total (SP group: *n* = 145, control group: *n* = 160), and the overall mean difference was not statistically significant (*Z* = 0.08; *p* = 0.94). On trial 5, short recall after interference, and recognition trials, three studies were compared (Grob et al., 1996; Doering-Silveira et al., 2005b; Barbosa et al., 2016). On trial five and the recognition trials, 166 participants (SP group: *n* = 84, control group: *n* = 82) were included. On the short recall trial after interference, the number was 167 (SP group: *n* = 85, control group: *n* = 82). None of the three overall mean differences were statistically significant (Trial 5: *Z* = 0.42; *p* = 0.68; Short recall after interference: *Z* = 0.09; *p =* 0*.*93; Recognition trials: *Z* = 0.20; *p =* 0.84).

#### Attention

Trail-Making Task. TMT-A was compared across three studies (Doering-Silveira et al., 2005b; Halpern et al., 2005; Barbosa et al., 2016). 276 participants (SP group: *n* = 131, control group: *n* = 145) were included, and the overall mean difference was not statistically significant, *Z* = 0.03 (*p* = 0.98).

#### Executive Functioning

Trail-Making task. TMT-B was also compared across three studies (Doering-Silveira et al., 2005b; Halpern et al., 2005; Barbosa et al., 2016). 276 participants (SP group: *n* = 131, control group: *n* = 145) were included, and the overall mean difference was not statistically significant, *Z* = 0.01 (*p =* 0*.*99).

Stroop. For the Stroop task word list, color list, and incongruent list, four studies were included (Doering-Silveira et al., 2005b; Halpern et al., 2005; Bouso et al., 2012; Barbosa et al., 2016). Bouso et al. (2012) assessed Stroop performance in ayahuasca users vs non-users in two different samples: people living in a jungle environment and people living in an urban environment. Since these samples were independent of each other, each sample (jungle vs urban) was treated as a separate study. Unlike the other studies, Halpern et al. (2005) and Doering-Silveira et al. (2005b) did not report the number of correct items but the overall time needed to complete a list. Furthermore, Doering-Silveira et al. (2005b) used the Victoria version of the Stroop task, a version that includes fewer items. Because of these differences, the overall standardized mean difference was calculated for the three trials instead of the overall mean difference. In each trial the number of participants added up to 514 (SP group: *n* = 257, control group: *n* = 257). Remarkably, the analysis indicated a better performance of the SP group in the incongruent list subtest (*Z* = 2.14; *p =* 0*.*03), while no significant difference emerged in the word list and color list subtests (*Z* = 0.19; *p =* 0*.*85 and *Z* = 1.18; *p =* 0*.*24).

#### Visuospatial Abilities

Rey–Osterrieth Complex Figure Task. On the ROCF, three studies were compared on the copy and delayed recall conditions (Doering-Silveira et al., 2005b; Halpern et al., 2005; Barbosa et al., 2016). For each condition, 276 participants (SP group: *n* = 131, control group: *n* = 145) were included, and none of the overall mean differences were statistically significant (Copy: *Z* = 1.13; *p =* 0*.*26; Delayed recall: *Z* = 1.46; *p =* 0*.*14).

#### Sensitivity Analyses

The results of our pre-planned sensitivity analyses are shown in Table 6. Restriction to only those studies with a NOS rating of seven or higher was not possible, as only one study (McGlothlin et al., 1969) fulfilled this criterion. Furthermore, all studies included in our main analyses already used matched control groups, rendering our second sensitivity analysis unnecessary. Restriction of analyses to the same SP was performed for the analyses on long delayed recall of the verbal learning tasks (VLT) and word, color, and incongruent lists of the Stroop task. No qualitative change was observed in the first three measures. However, the mean difference in the incongruent list of the Stroop task increased (*p =* 0*.*004). Analysis with exclusion of the study with the highest weight could be calculated for the same four tasks. Incidentally, the excluded studies were the same as in the previous sensitivity analysis.

**TABLE 6:** Overview of pre-planned sensitivity analyses.

Task–subtest	Unmodified analysis	Matched control groups	Restriction to same serotonergic psychedelics	Exclusion of study with greatest weight
VLT–Trial 5	3 pairs MD 0.24 [-0.87, 1.35], *p =* 0*.*68, I^2^ = 69%	N/A^1^	N/A^1^	N/A^2^
VLT–Short recall after interference	3 pairs MD -0.05 [-1.11, 1.02], *p =* 0*.*93, I^2^ = 57%	N/A^1^	N/A^1^	N/A^2^
VLT–Long delayed recall	4 pairs MD 0.03 [-0.59, 0.64], *p =* 0*.*94, I^2^ = 43%	N/A^1^	3 pairs (Ayahuasca) MD -0.13 [-1.01, 0.76], *p* = 0.78, I^2^ = 43%	3 pairs MD -0.13 [-1.01, 0.76], *p* = 0.78, I^2^ = 43%
VLT–Recognition	3 pairs MD -0.04 [-0.40, 0.32], *p =* 0*.*84, I^2^ = 31%	N/A^1^	N/A^1^	N/A^2^
TMT-A	3 pairs MD -0.03 [-1.99, 1.94], *p =* 0*.*98, I^2^ = 9%	N/A^1^	N/A^2^	N/A^2^
TMT-B	3 pairs MD -0.03 [-7.36, 7.29], *p =* 0*.*99, I^2^ = 57%	N/A^1^	N/A^2^	N/A^2^
Stroop–Word list	5 pairs MD 0.05 [-0.43, 0.52], *p =* 0*.*85, I^2^ = 86%	N/A^1^	4 pairs (Ayahuasca) MD 0.14 [-0.42, 0.69], *p* = 0.63, I^2^ = 86%	4 pairs MD 0.14 [-0.42, 0.69], *p* = 0.63, I^2^ = 86%
Stroop–Color list	5 pairs MD 0.25 [-0.17, 0.68], *p =* 0*.*24, I^2^ = 82%	N/A^1^	4 pairs (Ayahuasca) MD 0.34 [-0.15, 0.84], *p* = 0.18, I^2^ = 82%	4 pairs MD 0.34 [-0.15, 0.84], *p* = 0.18, I^2^ = 82%
Stroop–Incongruent list	5 pairs MD 0.38 [0.03, 0.72], *p =* 0*.*03, I^2^ = 73%	N/A^1^	4 pairs (Ayahuasca) MD 0.49 [0.16, 0.83], *p* = 0.004, I^2^ = 60%	4 pairs MD 0.49 [0.16, 0.83], *p* = 0.004, I^2^ = 60%
ROCF–Copy	3 pairs MD 0.30 [-0.22, 0.83], *p =* 0*.*99, I^2^ = 57%	N/A^1^	N/A^2^	N/A^2^
ROCF–Delayed recall	3 pairs MD 1.03 [-0.35, 2.42], *p =* 0*.*14, I^2^ = 0%	N/A^1^	N/A^2^	N/A^2^

*N/A* = analysis not available; ^1^unmodified analysis already fulfilled this criterion; ^2^sample size <three. *VLT*, Verbal Learning Task; *TMT*, Trail Making Test; *ROCF*, Rey-Osterrieth Complex Figure Test

## Discussion

In our systematic review and meta-analysis on the relationship between repeated use of serotonergic psychedelics (SPs) and neuropsychological performance, we report the following findings: 1) The vast majority of participants stemmed from studies specifically investigating ayahuasca (6 studies, *n* = 343), followed by five studies investigating LSD (*n* = 101), one study that investigated peyote (*n* = 61), and another study that did not specify the investigated SP (*n* = 34). No studies were available on psilocybin, 5-MeO-DMT, or any other specific SPs. 2) All of the included studies had considerable methodological limitations: No study was rated as being of high quality, and 10 out of 13 studies did not sufficiently match SP-users to controls on their use of other, non-psychedelic psychoactive substances. 3) The three studies which applied a rigorous matching procedure but without reaching a high rating of study quality covered three SPs (peyote, ayahuasca, LSD) and reported conflicting results. 4) Our qualitative review did not detect a clear pattern of neuropsychological consequences related to SP use across different types of SPs. However, one study found impaired neuropsychological performance in LSD users, while several studies associated ayahuasca use with increased neuropsychological performance. 5) Finally, in our quantitative analysis, SP users outperformed their controls in a task assessing executive functioning (Stroop task). However, as only one study included in the meta-analysis successfully controlled for confounding factors such as substance use, our findings should be considered as preliminary.

In the next sections, we will discuss the included studies which can be divided into two groups: 1) studies from 1969 to 1983, which almost exclusively investigated users of the semi-synthetic ergoline SP LSD, which was by far the most commonly used SP then and still is today (Winstock et al., 2021), and 2) studies from 1996 to 2020, which investigated two plant-derived SPs, namely ayahuasca (containing the tryptamine DMT) and peyote (with the phenethylamine mescaline as psychedelic ingredient). Notably, the first group includes mostly recreational users also prone to using other psychoactive substances, whereas the second group includes members of communities using SPs in religious or ritualized settings, with an overall lower use of other substances.

### Studies From 1969 to 1983: Lysergic Acid Diethylamide

In the first identified period of research, LSD was by far the most intensively used SP, which is reflected in the neuropsychological studies predominantly focusing on LSD. As mentioned above, the majority of studies were of insufficient quality and did not adequately control for other, non-psychedelic substances. Arguably the first controlled study investigating neuropsychological consequences of SP use was conducted by Cohen and Edwards (1969), who compared 30 users of LSD (with a median of 70 LSD exposures, see Table 1) to 30 controls matched on gender, age, educational level, and socio-economic background and found that LSD users showed lower attentional and visuo-spatial performance (see Table 1 for details). However, the LSD group had not only taken LSD more often than the control group, but had also used more cannabis, amphetamines, barbiturates, heroin, and cocaine. Based on the results of this study, McGlothlin et al. (1969) compared 16 participants with a history of LSD exposure (partly in a therapeutic setting; median 75 LSD exposures) and compared these with 16 controls matched on age, gender, education, occupation, and the number of people who had received psychotherapy (without LSD). The authors report reduced performance in a categorical task for LSD users. Even though this study received the highest rating regarding study quality, it did not include sufficient matching in terms of substance use. Four participants of the control groups had previously used cannabis ten or more times, while in the LSD group eight had done so. Additionally, six participants in the LSD group had low to moderate use of opiates, sedatives and stimulants, but the history of use of those substances was not quantified (McGlothlin et al., 1969, p.2). In a similar fashion, Wright and Hogan (1972) compared 20 frequent recreational LSD users (mean number of LSD exposures = 29.3) with 20 controls, matched for gender, age, education, and intelligence, and found LSD users to perform better on one but worse on another subtest of the WAIS. It was reported that members of the control groups had not used any drugs, while of the 20 members of the LSD group, 19 reported cannabis use, 10 methamphetamine use, five opium use, and a few other substances (cocaine, various stimulant medications, various sedatives or opiates) had each been used by less than five participants. Another study (Culver and King, 1974) aimed to compare LSD users with non-users, recruiting 14 LSD users with additional cannabis use (and a median of 17 LSD experiences), 14 cannabis users, and 14 controls without a history of LSD or cannabis use. Additionally, they matched participants in all three groups according to verbal aptitude, mathematical ability, and personality profiles, and excluded participants for regular use of other substances. Furthermore, the cannabis group and the LSD group reported similar amounts of lifetime cannabis and alcohol use. In their investigation, they found that the LSD users performed worse than the cannabis users in tests of attentional performance and executive functioning (trials A and B of the Trail Making Test), indicating that this difference might be due to the use of LSD.

Apart from this apparent negative association of LSD use with neuropsychological functioning, LSD is the substance for which association with Hallucinogen Persisting Perception Disorder (HPPD) has most commonly been reported–even if this might reflect the overall high frequency of LSD use when compared to other SPs, and not a relative risk (Martinotti et al., 2018). Trying to investigate attentional performance of SP users with flashbacks, Matefy et al. (1979) compared 29 SP users with flashbacks, 25 SP users without flashbacks, and 23 controls without any substance use on a simple reaction-time task. All three groups were similar in terms of age, sex, hobbies, education, and their father’s education. SP users showed an increased reaction time across five measures. However, in an earlier publication, the authors report that the two SP groups were using cannabis in addition to SPs and had previously used sedatives, stimulants, cocaine, and heroin (Matefy et al., 1978). Standing out from these previous lines of research dealing with mostly healthy recreational users, Vardy and Kay (1983) compared neuropsychological performance in 29 patients who had been hospitalized because of psychotic symptoms following LSD use with 29 patients with schizophrenia without any history of LSD or other drug use. The authors report no differences in their neuropsychological tests. However, they acknowledge that the patients with a history of LSD use also report “more general drug experience”, without clarifying or quantifying history of drug use (Vardy and Kay, 1983, p. 2). Due to its focus on patient populations, this study is markedly different from the other studies discussed here, and its results cannot be generalized to healthy participants.

### Studies From 1996 to 2020: Ayahuasca and Peyote

After this early period of LSD research, no further studies have been published dealing with the neuropsychological consequences of regular LSD use. Given the high overall polyvalent substance use of LSD users associated with the aforementioned methodological problems, studies from the 1990s started to investigate religious or ethnic groups ritually using specific SPs but having low use of other substances. One of these studies, and indeed the only investigation of a phenethylamine SP included in our review, Halpern et al. (2005) compared 61 religious users of peyote (with a median of 300 peyote experiences) with 36 former patients suffering from alcohol use disorder and 79 controls with minimal substance use. To control for other substance use, participants were only included if they reported lifetime use of cocaine, stimulants, opioids, sedatives, other SPs than peyote, or inhalants less than ten times, and less than 100 lifetime occasions of cannabis use. With these rigorous controls in place, the authors report no differences in any of the eight neuropsychological tests between the peyote group and the control group.

Investigating a different kind of religious SP use, several studies have studied consequences of regular ayahuasca use in members of syncretic Brazilian churches that regularly use ayahuasca as a sacrament in religious rituals. Among these, the church União do Vegetal (UDV) has been considered particularly useful for research (Grob et al., 1996; Doering-Silveira et al., 2005b; Barbosa et al., 2016), as UDV members are required to abstain from the use of any other substances, including alcohol and cannabis. Consequently, UDV members show very high lifetime use of ayahuasca and usually comparably low use of other substances. In a first study, 15 male members of the UDV (with at least 240 ayahuasca experiences, see Table 1) were compared with 15 male controls, who were closely matched to the ayahuasca group in terms of age, ethnicity, marital status, and level of education (Grob et al., 1996). The authors found that the ayahuasca users performed better on one measure of verbal memory. Matching for the use of other substances was not entirely successful: While the members of the ayahuasca group had been church members for at least 10 years and therefore abstinent from other substances for that time, eleven members reported moderate to severe alcohol use and five reported a history of cocaine and amphetamine use before their engagement with the church. Additionally, two members of the control group reported a current alcohol use disorder. Another study (Doering-Silveira et al., 2005b) investigated the differences in neuropsychological performance between 40 adolescent members of the UDV church (with at least 24 ayahuasca experiences) and 40 adolescent non-users matched to the ayahuasca group in terms of age, gender, race, and educational level, also reporting an increased performance for ayahuasca users in verbal memory. Still, a previous investigation of these two groups showed that they differed significantly in the prevalence of different psychoactive substance use, mainly alcohol, amphetamine, and solvents (Doering-Silveira et al., 2005a). Furthermore, Barbosa et al. (2016) investigated another 30 members of the UDV church (with a median of 150 ayahuasca exposures) and matched them to a control group without a history of ayahuasca use of 27 in terms of membership in a religious organization, age, and gender. In this study, the authors likewise report an increased verbal learning performance in ayahuasca users. Similarly to previous research with UDV members (Grob et al., 1996), the authors found that the members of the ayahuasca group had a higher lifetime exposure to other substances, in this case alcohol and cannabis. Additionally, the control group had used significantly more alcohol in the past month compared to the UDV group.

In a larger study with members of the Santo Daime ayahuasca church, which has less intense restrictions on the use of other substances, Bouso et al. (2012) further investigated neuropsychological consequences of ayahuasca use. They recruited ayahuasca users (with 260–1,440 experiences) from a community within the Amazon rain forest (*n* = 56) as well as users from an urban setting (*n* = 71) and compared these groups to controls from similar settings (*n* = 56 from a town close to the Amazon group, and *n* = 59 from the same city as the urban group), finding a higher level of executive functioning in ayahuasca users. However, in a previous study using the same groups, the authors reported that both ayahuasca groups had more past-month cannabis use and higher lifetime amphetamine and cocaine use than the control groups (Fábregas et al., 2010). Finally, the most recent study included in our review compared 30 ayahuasca users (with a median of 10 exposures) with 30 non-users in Estonia (Kaasik and Kreegipuu, 2020), with no significant differences in intelligence. Similarly to Bouso et al. (2012), they also found that their ayahuasca-using participants had used more cannabis than their control group. Across the studies conducted in religious settings, it becomes clear that even those subjects are often not free from a history of polysubstance use, and that even recruitment from churches with presently abstinent users does not always allow for successful control in terms of co-occurring substance use.

A single study, Bouso et al. (2015), explicitly required participants in both study and control groups to report lifetime cannabis use on less than 20 occasions and lifetime use of other substances on less than ten occasions. In the study, 22 ayahuasca users (with a mean of 123 ayahuasca exposures) outperformed 22 controls on tasks related to working memory (two-back task) and executive functioning (task-switching).

### Ayahuasca and Improved Neuropsychological Performance

This association between ayahuasca use and improved executive control is partially supported by our meta-analysis, which showed that ayahuasca users consistently performed better in the inhibitory control section of the Stroop task. Taken together with the results of the well-controlled study by Bouso et al. (2015), these results may hint at a beneficial effect of ayahuasca use on executive functioning. This conclusion is surprising insofar as regular use of other substances such as alcohol, cocaine, or methamphetamine associates with impairments in executive functioning (Verdejo-García et al., 2006; Fernández-Serrano et al., 2009; Le Berre et al., 2010; Nestor et al., 2011). One explanation for this proposed effect of ayahuasca use could be related to its pharmacological mechanism. Tryptamines, such as DMT, show a higher binding affinity to the 5-HT_1a_R than the 5-HT_2a_R most SPs bind to (Fantegrossi et al., 2008; Winter 2009). Furthermore, tryptamines involve more 5-HT_1a_R agonism than phenethylamines (Halberstadt and Geyer, 2011). The 5-HT_1a_R has been shown to play a role in cognitive control in both humans (Langenecker et al., 2019) and animals (Baba et al., 2015). In addition, use of a 5-HT_1a_ agonist over the course of 6 weeks has been shown to increase executive functioning in patients with schizophrenia (Sumiyoshi et al., 2001). Regular administration of ayahuasca, an agonist at the 5-HT_1a_R may induce similar improvements. This hypothesis could also explain why a similar pattern was not found in well-controlled studies with users of LSD (Culver and King, 1974) or peyote (Halpern et al., 2005), as both substances differ significantly from members of the tryptamine class in their receptor binding profiles (Ray, 2010). Another explanation for these results might be related to the involvement of specific brain structures. It has been reported that performance in the Stroop task was associated with activation of the anterior cingulate cortex (ACC) (Peterson et al., 1999; Ridderinkhof et al., 2004; Tolomeo et al., 2016) and Bouso et al. (2015) showed that use of ayahuasca was associated with higher cortical thickness in the ACC. We hypothesize that structural differences in specific brain regions between ayahuasca users and non-users might contribute to the improved performance in the Stroop task in users, however, this interpretation is highly preliminary and requires further research. Finally, since behaviors that improve neuronal plasticity are associated with improved cognitive performance (Greenwood and Parasuraman, 2010), and SPs have been shown to induce structural and functional plasticity (Marinova et al., 2017; Ly et al., 2018), it is not surprising that SP use in some domains might be associated with neuropsychological improvement.

**FIGURE 2:**
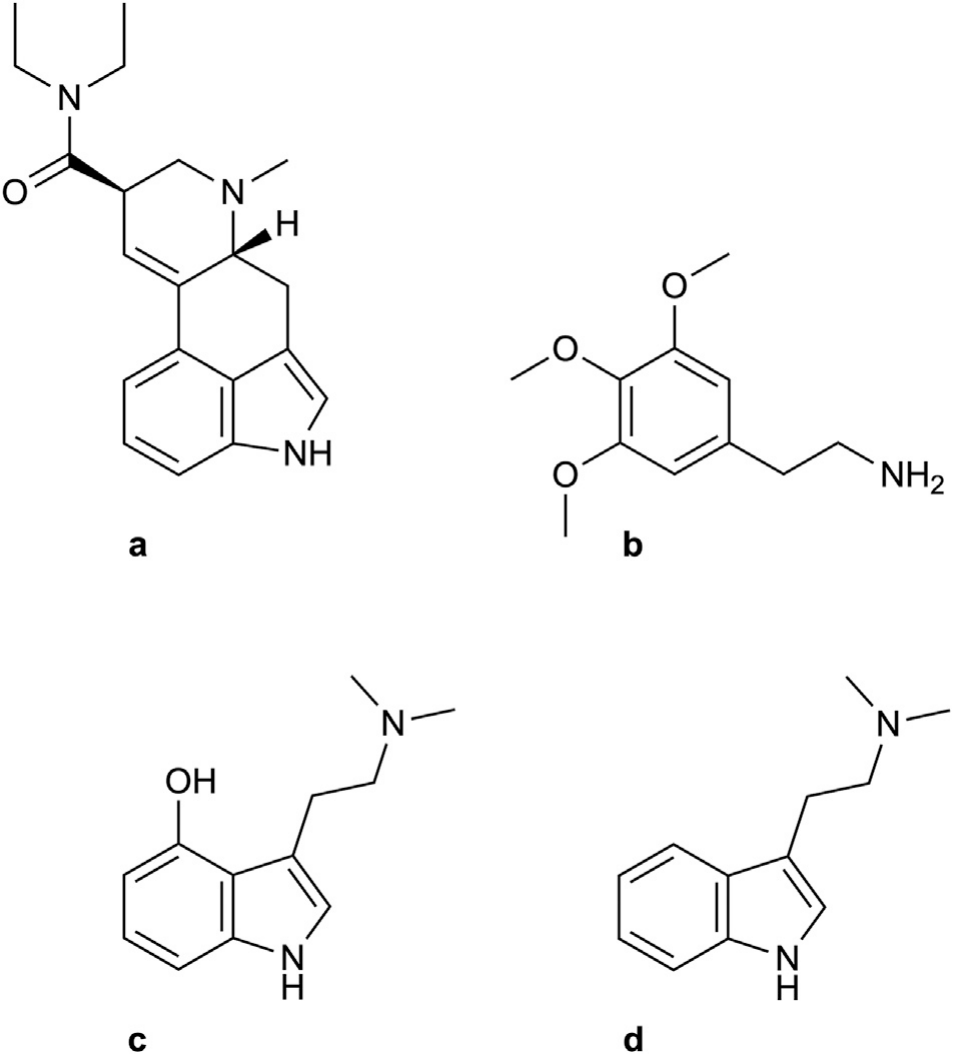
Structural formula of serotonergic psychedelics investigated in studies (sample size). A) LSD (n=135), B) Mescaline (n=61), C) psilocin (the active metabolite of psilocybin, n=0), D) DMT (serotonergic psychedelic ingredient of ayahuasca, n=343).

Although these may be intriguing hypotheses, it bears reiterating that most research in this field is cross-sectional, not allowing any conclusions on causality. Surprisingly, even though psilocybin has been the most intensively investigated SP in humans in the past 25 years (Johnson and Griffiths, 2017), we could not find any studies assessing the neuropsychological consequences of its use. As psilocybin and its active metabolite psilocin show a similar binding affinity to the 5HT1a receptor (Rickli et al., 2016) and are structurally very similar to DMT (see Figure 2), the main psychoactive component of ayahuasca, which was investigated in several of our included studies, we speculate that their neuropsychological consequences could be of a similar nature. However, studies with well-matched control groups are necessary before any conclusions about the neuropsychological effects of psilocybin can be drawn. Specifically, there is a need for studies taking strong measures to control for potential confounding factors, especially with regard to substance use. Matching the control group and exposure group should be of highest priority when designing a study to establish consequences of repeated use. Since all modern studies included in our review have been conducted in ritual users of mescaline or ayahuasca, future research should take care to include users of psilocybin, as this is one of the most frequently used SPs in recreational settings (Krebs and Johansen, 2013) and has been most extensively investigated in clinical studies (Bogenschutz and Ross, 2018). Notably, the relevance of our findings regarding SP-assisted therapy remains limited, as users in our studies mostly used SPs repeatedly in recreational or traditional context, without any psychotherapeutic support and without knowledge on the exact concentration and purity of substances. Clinical use of SPs involves pharmacologically pure substances of a defined dosage, whereas in recreational use, the consumed substance is often not reliably defined (Hirschfeld et al., 2021) and even in traditional use, dosage and composition might often underlie strong variations (Gaujac et al., 2013). Nevertheless, many of the participants in the identified studies reported excessive lifetime use of SPs, by far exceeding SP exposition of patients in clinical studies of SP-assisted therapy, where substances are administered only very few times, never reaching the lifetime use of participants in the reported studies. In conclusion, as heavy use was not associated with decreases in neuropsychological outcome, it appears unlikely that negative effects would be found when SPs are administered only rarely, as it is the case for SP-assisted psychotherapy.

### Limitations

As mentioned above, a major limitation of this review is the difficulty of controlling for the use of other psychoactive substances evident in the included studies. Implementing this control is challenging, as the majority of SP users report use of additional psychoactive substances, especially cannabis (Pisano et al., 2017). In trying to restrict the studies to participants taking SPs exclusively, some studies recruited their samples in groups of a specific religious background who use SPs as part of their religious practice, leading to a very narrow selection of participant demographics. In fact, nearly all studies in this field conducted after 1990 are limited to the use of ayahuasca or peyote in ritualized/religious settings.

Although substances belonging to the group of SPs may overlap regarding phenomenological aspects of associated psychedelic experiences, generalization of the findings of possible beneficial effects of ayahuasca to the whole group of SPs (and even to all tryptamine psychedelics) remains problematic. This is starkly illustrated by the fact that one well-controlled study involving LSD users (Culver and King, 1974) reported diminished performance in tasks dealing with executive functioning and attention, and Halpern et al. (2005) reported no impairments or improvements in peyote users. Furthermore, the analyzed studies in our meta-analysis of Stroop task performance (Grob et al., 1996; Doering-Silveira et al., 2005b; Bouso et al., 2012) did not entirely succeed in controlling for other substance use. Therefore, it cannot be ruled out that the better performance in the ayahuasca-using group might also reflect effects of other substances, or other non-substance related differences between groups.

## Conclusion

While use of SPs is generally considered to be relatively safe when carried out in controlled clinical settings, the present review indicates that reliable data on neuropsychological consequences of repeated SP use is scarce. Notably, we did not find any studies assessing the neuropsychological consequences of psilocybin use, which is the SP investigated in most clinical settings nowadays. It appears that controlling for use of other psychoactive substances or other confounding variables between SP-users and non-users is very difficult and often unsuccessful, as polyvalent use is prevalent even in subjects who ritually use SPs. Interestingly, we found that in some well-controlled studies, LSD use was associated with lower task-switching performance, and ayahuasca use was associated with a higher performance in inhibitory control, whereas peyote use was not related to any differences in neuropsychological performance. Future research in this field should aim to clarify if these differences are a reflection of differences in pharmacological action.

## Data Availability

The raw data supporting the conclusions of this article will be made available by the authors, without undue reservation.
