# Supplementary material for: Neuropsychological Functioning in Users of Serotonergic Psychedelics – A Systematic Review and Meta-Analysis
Source: Front Pharmacol. 2021 Sep 16;12:739966. doi: 10.3389/fphar.2021.739966 (PMC8481924; doi:10.3389/fphar.2021.739966)

Supplementary Material

# Supplementary Methods section

The following PICOS criteria were used for our search:

**Population***:* Studies with healthy participants and various patient populations were included.

**Intervention***:* To be included, a paper had to be either a case report of neuropsychological dysfunction after SP use, a qualitative study dealing with neuropsychological abilities of people who frequently use SPs, an experimental study in which subjects were given frequent dosages of a SPs and administered a neuropsychological test battery, or an observational study that performed neuropsychological assessment on a sample of SP users. All assessments had to be conducted with a test instrument yielding quantitative data.

**Comparisons***:* Studies were selected if they compared the scores of the SP users to a placebo group, a control group, or to normative data.

**Outcomes***:* To be considered the article had to report the results of at least one measure, assessing at least one neuropsychological domain.

**Study type***:* Case reports, qualitative studies of frequent users of SPs, observational studies of frequent users of SPs, or experimental human studies with frequent administration of SPs, published in peer-reviewed journals were included. We excluded animal research, review papers, opinion pieces, letters to the editors (unless a case report was presented as such), and books.

# Supplementary Figures and Tables

**Supplementary Table 1***.* Included search terms related to psychedelic substances and neuropsychological testing or domains.

|  | **Search Terms** |
| --- | --- |
| **Psychedelic terms** | “2c-e”, “4-bromo-2,5-dimethoxyamphetamine”, “2c-b”, “psilocin”, “2,5-dimethoxy-4-methylamphetamine”, “nbome”, “iboga”, “bufotenin”, “ayahuasca”, “ibogaine”, “n,n-dimethyltryptamine”, “psilocybin”, “mescaline”, “peyote”, “dmt”, “lysergic”, “lsd”, “hallucinogen”, “psychedelic” |
| **Neuropsychological terms** | “amnes*”, “neuropsychological”, “intelligence”, “attention”, “cognitive”, “learning”, “memory”, “neurocognitive”, “neuropsychiatric”, “psychological”, “psychometric”, “recall”,” recognition”, “executive function”, “aphasia”, “agnosia”, “apraxia”, "wisconsin card", "boston naming", "rey osterrieth", “mmse”, “stroop”, "digit span", "trail making", "word list", “fluency” |

**Supplementary Table 2***.* Rating of study quality according to the Newcastle-Ottowa-Scale.

|  | **Selection** | | | | **Comparability** | | **Exposure** | | | **Total score (max. 9)** |
| --- | --- | --- | --- | --- | --- | --- | --- | --- | --- | --- |
| Barbosa et al., (2016) | q1 | q2 | q3 | q4 | q1 | q2 | q1 | q2 | q3 | 5 |
| Bouso et al., (2012) | q1 | q2 | q3 | q4 | q1 | q2 | q1 | q2 | q3 | 4 |
| Bouso et al., (2015) | q1 | q2 | q3 | q4 | q1 | q2 | q1 | q2 | q3 | 4 |
| Cohen & Edwards, (1969) | q1 | q2 | q3 | q4 | q1 | q2 | q1 | q2 | q3 | 5 |
| Culver & King, (1974) | q1 | q2 | q3 | q4 | q1 | q2 | q1 | q2 | q3 | 6 |
| Doering-Silveira et al., (2005) | q1 | q2 | q3 | q4 | q1 | q2 | q1 | q2 | q3 | 6 |
| Grob et al., (1996) | q1 | q2 | q3 | q4 | q1 | q2 | q1 | q2 | q3 | 6 |
| Halpern et al., (2005) | q1 | q2 | q3 | q4 | q1 | q2 | q1 | q2 | q3 | 6 |
| Kaasik & Kreegipuu (2020) | q1 | q2 | q3 | q4 | q1 | q2 | q1 | q2 | q3 | 3 |
| Matefy, Hayes & Hirsch, (1979) | q1 | q2 | q3 | q4 | q1 | q2 | q1 | q2 | q3 | 4 |
| McGlothlin, Arnold, & Freedman, (1969) | q1 | q2 | q3 | q4 | q1 | q2 | q1 | q2 | q3 | 7 |
| Vardy & Kay, (1983) | q1 | q2 | q3 | q4 | q1 | q2 | q1 | q2 | q3 | 4 |
| Wright & Hogan, (1972) | q1 | q2 | q3 | q4 | q1 | q2 | q1 | q2 | q3 | 5 |

*Note:* Each cell represents one question of the Newcastle-Ottowa-Scale. A red cell indicates that the criterion in question was not fulfilled, while a green cell indicates fulfillment of the criterion.

## Supplementary Figures

**Supplementary Figure 1.** Forest plots for Rey-Osterrieth Complex Figure Task. A) Copy trial B) Delayed Recall trial


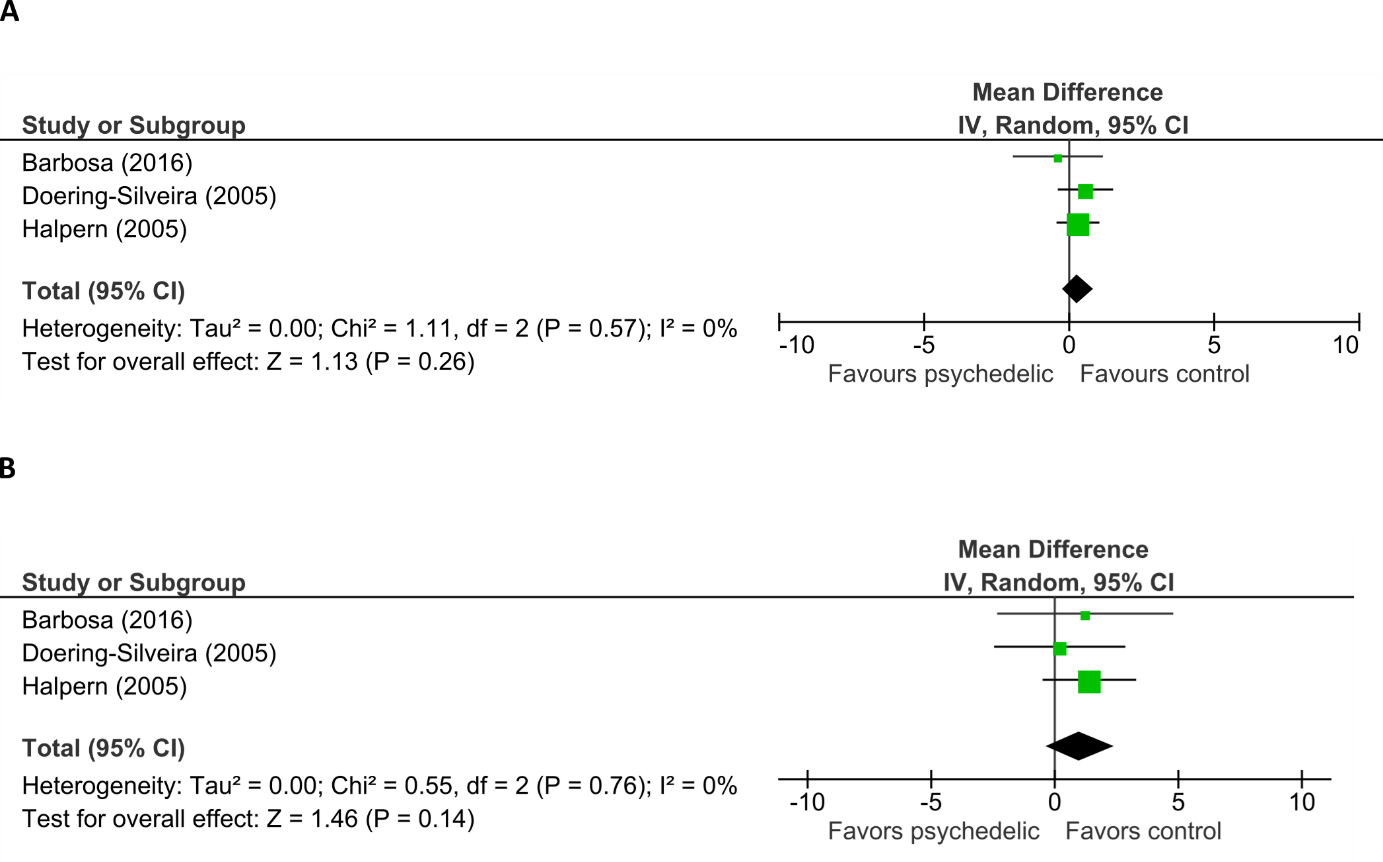


**Supplementary Figure 2.** Forest plots for the Trail Making Test. A) Trail A B) Trail B


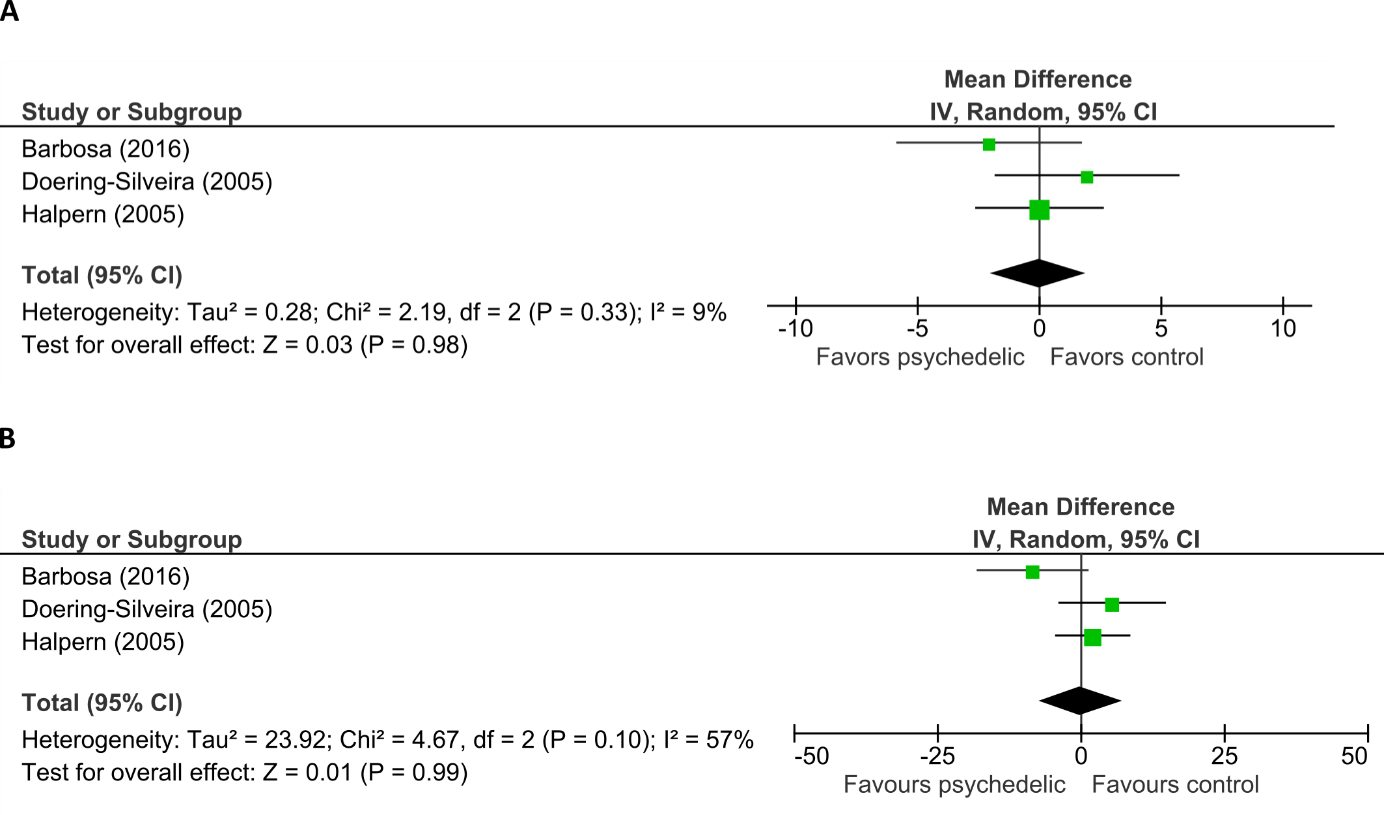


**Supplementary Figure 3**. Forest plots for the Stroop task. A) Word trials B) Color trials C) Incongruent trials


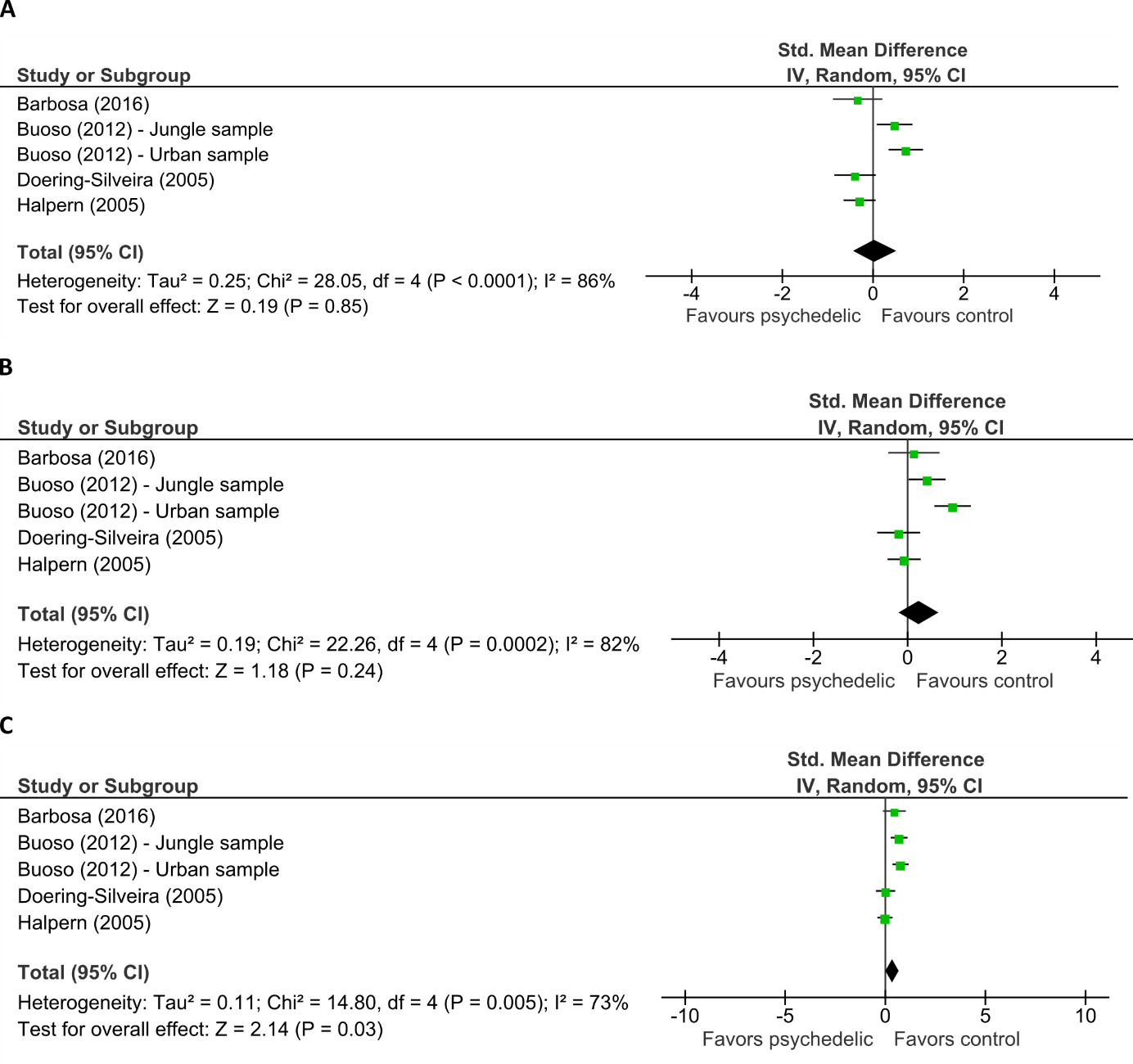


**Supplementary Figure 4**. Forest plots for the Verbal Learning tasks. A) Trial 5 B) Short Recall after Interference C) Recognition D) Long Delayed Recall


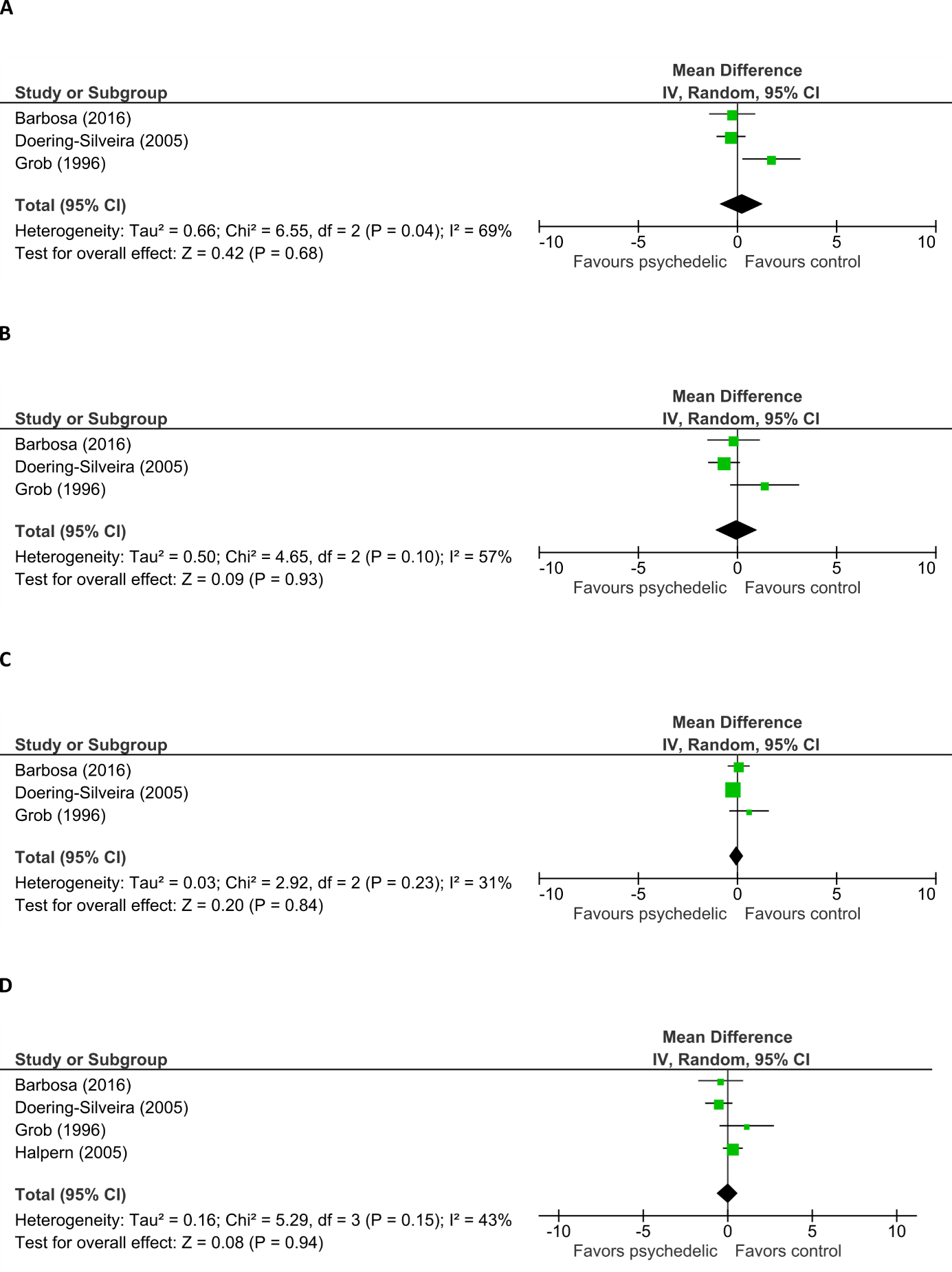

Supplement: Supplementary file 1 [file DataSheet1.docx]
